# Internet-based Cognitive-behavioral therapy (CBT) for depressive symptomatology in individuals with type 1 diabetes (WEB_TDDI1 study): A randomized controlled trial protocol

**DOI:** 10.1371/journal.pone.0274551

**Published:** 2022-09-20

**Authors:** Mónica Carreira, María Soledad Ruiz de Adana, José Luis Pinzón, María Teresa Anarte-Ortiz

**Affiliations:** 1 Department of Personality, Assessment and Psychological Treatment, Biomedical Research Institute of Malaga (IBIMA), University of Malaga, Málaga, Spain; 2 Clinical Management Unit of Endocrinology and Nutrition, Biomedical Research Institute of Malaga (IBIMA), Regional University Hospital, University of Malaga, Málaga, Spain; 3 Clinical Management Unit of Endocrinology and Nutrition, Biomedical Research Institute of Malaga (IBIMA), Virgen de la Victoria University Hospital, University of Malaga, Málaga, Spain; Public Library of Science, UNITED KINGDOM

## Abstract

**Background:**

Professionals need adequate tools to help patients with diabetes and depression. Although web programs integrating cognitive-behavioral therapy with diabetes education have shown good results, no similar approach has been performed as yet in Spain. The objective is to develop an Internet-based program for the treatment of mild-moderate depressive symptomatology in individuals with type 1 diabetes (WEB_TDDI1 study) based on Cognitive-behavioral therapy (CBT) and assess its results.

**Methods:**

A 2-arm randomized controlled trial will be conducted. Adults with type 1 diabetes and mild-moderate depressive symptoms will be screened to participate in the study and randomly assigned to either the treatment group (TG) that will use a Web-based application for a specific 9-week intervention in depression and type 1 diabetes or the control group (CG) that will be on the waiting list during that time.

**Results:**

Data on the primary variable (depressive symptoms) and secondary variables (treatment-related distress, anxiety, fear of hypoglycemia, quality of life, treatment adherence, coping strategies and glycemic control) will be collected from the TG at the beginning/baseline, at the end of treatment and at 3, 6 and 12 months after treatment. The CG will be assessed at the beginning and at the end of the TG intervention. On completion of the program by the TG, the treatment will then be carried out in the CG.

**Conclusions:**

The new web application developed is expected to be effective for the treatment of mild-moderate depressive symptoms in adults with type 1 diabetes, reducing depressive symptoms and improving the rest of the analyzed variables.

**Trial registration:**

Registry: NCT03473704 (March 21, 2018); ClinicalTrials.gov.

## Introduction

The risk of depression is 3 times higher in individuals with type 1 diabetes than in the general population [1]. The presence of both diseases increases the number of complications resulting from diabetes [2, 3], decreases adherence to diabetes treatment [4, 5] and increases healthcare costs compared to individuals with diabetes who do not have depression [6, 7]. Consequently, early detection and proper treatment is especially important. However, only 25% of people with diabetes and depression are identified during their visit to the doctor [8] and, therefore, 75% may not have access to correct treatment.

According to a study conducted by Anarte et al., [9] which examined the accuracy of the physician in detecting depression in patients with type 1 diabetes, 25% of patients with depression were not identified. The difficulty of identifying this disorder in the population with diabetes results in treatment being delayed or possibly not even provided. Petrak et al. [10] stress the importance of the correct treatment of depression for good diabetes self-care, as individuals with diabetes may adhere to their treatment more easily if they are in a good mood.

Research confirms that treatment of depression in people with diabetes is effective [11, 12]. The interventions that have been carried out for the treatment of depression in diabetes consist of psychological interventions, psychopharmacological treatments, dietary treatments and physical activity. Psychological intervention has shown better results than usual care and waiting list in reducing positive symptoms [10] Cognitive-behavioral therapy (CBT) is one of the psychological interventions used. This therapy is an effective treatment for adults with depression [13].

The evidence available through 4 meta-analyses of the main randomized controlled trials [14–17] and one systematic review [18] indicates that CBT is effective in treating depression in individuals with diabetes.

With the rise of new technologies among the population, new forms of treatment through the Internet are now possible, offering great advantages over traditional treatments, such as accessibility, reducing the stigma of diabetes and therapist interaction time while still maintaining effectiveness [19]. Internet-based treatment has been shown to be effective for depression [20], as it has been for other chronic diseases [21], although additional research on its long-term effect is needed [22]. Two pioneering platforms for the general population that have obtained positive results are MoodGYM and Beating the Blues [23, 24]. In Spain, an Internet platform based on CBT called Smiling is Fun has been created for primary care, demonstrating a benefit when implemented together with usual care [25]. The use of Internet-based CBT has also been shown to be effective in the treatment of depressive symptoms [26].

In diabetes, the use of new technologies allows easy access to a greater number of users [27]. Application of these technologies has been shown to be a good tool in the treatment of depression yielding positive results [28]. For the treatment of depression in people with diabetes, several authors have designed web programs incorporating CBT. In the first study carried out for this purpose, van Bastelaar et al. [29–31] designed and administered a specific treatment program for people with diabetes that comprised 8 sessions comparing the results with those of a control group that received no treatment. They obtained positive results in depression and specific distress but not in glycemic control. Based on the work of van Bastelaar et al., [29–31] Nobis et al. [32–34] developed a web-based intervention with telephone support for adults with diabetes. In this case, the results of the 6-session web treatment (plus 2 optional sessions) were compared with a psychoeducation program. Again, there was an improvement in depression and diabetes-specific distress in the intervention group, although there were no changes in glycemic control. The benefits found were maintained at the 6-month follow-up.

Other authors have assessed the effectiveness of Web-based CBT treatment programs for the general population in individuals with diabetes. Robins et al. [35] designed a study in which they compared a 6-session Web-based treatment for the general population with a control group treated with usual treatment, obtaining positive results in mood and diabetes-specific distress, among others, but not in glycemic control. These results were maintained after 3 months of follow-up [36]. Clarke et al. [37] designed a study to evaluate the effect of a general population CBT program (myCompass) on the well-being of young people with type 1 diabetes. The results of this study have not yet been published. Nevertheless, they analyzed the results of this 7-session program in a pre-post study without a control group in a population with type 1 and type 2 diabetes and found significant improvement in depressive symptoms and specific distress, among other variables, although data on glycemic control were not collected [38] Thus, these treatment programs could also be offered as a possible avenue of intervention in people with diabetes.

According to previous studies, 21.7% of people with type 1 diabetes in our population have depressive symptoms [39]. There is a clear need to provide professionals with adequate tools to help people with this problem.

Based on the results obtained in previous studies, we have developed an Internet-based program for the treatment of mild-moderate depressive symptomatology for individuals with type 1 diabetes (WEB_TDDI1 study). In this study, we have created a specific program for people with type 1 diabetes including specific modules aimed at improving depressive symptomatology in this population and, indirectly, other variables related to depression in diabetes such as coping, anxiety, distress, and fear of hypoglycemia, which affect glycemic control, adherence to diabetic treatment and quality of life. This program has been designed specifically for individuals with type 1 diabetes because there are differential characteristics between type 1 and type 2 diabetes that should be considered separately [40] There are also important differences in their association with depression, suggesting that the associations are mediated by different underlying mechanisms [41] This article presents the design of this program and the methodology for its development and assessment.

The main objective of this project is to apply an internet-based program for the treatment of depression specific to individuals with type 1 diabetes. The program, designed by our research team (WEB_TDDI1 study), will be implemented in a sample of patients with type 1 diabetes and mild-moderate depressive symptoms in the province of Malaga (Spain).

## Material and methods

### Study design and participants

This study is a longitudinal randomized control-group pretest-posttest clinical trial (S1 Table). Patients with type 1 diabetes who present depression (mild to moderate) and meet the inclusion criteria will be randomized through a computer program to 2 groups. The treatment group (TG) will receive the 9-week Web-based treatment, while the control group (CG) will comprise patients on the waiting list. Evaluations in the TG will be conducted at the beginning of the program (baseline), at completion and during follow-up (3, 6, and 12 months) after treatment. For ethical reasons, the CG will receive the Web-based treatment once the TG intervention has been completed. The CG will thus be assessed at the beginning and at the end of the TG treatment. At this point, the CG will begin treatment, provided the participants still meet the eligibility criteria, and will have their corresponding evaluations. The assessments will be conducted in person.

The participants will be individuals with type 1 diabetes from the province of Malaga (Spain) who meet the following criteria:

○ Inclusion criteria: medical diagnosis of type 1 diabetes ≥ 1 year; over 18 years of age; psychological diagnosis of mild/moderate major depressive disorder, dysthymia or depressive symptomatology; no concomitant pharmacological treatment that could modify blood glucose values or depressive symptomatology; no psychological treatment at the present time; absence of the following: chronic renal failure, impaired liver function tests, active thyroid disease (except correctly substituted hypothyroidism), current pregnancy or acute ketosis decompensation at the beginning of the study; Internet access.
○ Exclusion criteria: type 2 diabetes; women who are pregnant or planning to become pregnant; severe macrovascular or microvascular complications; diagnosis of major depressive disorder with risk of suicide; non-collaboration (no signed informed consent); presenting a disabling psychiatric disorder; psychosis; no Internet access.

All participants in this study must give their informed consent. The ethical standards of care in the provision of telepsychology services of the General Council of Psychology of Spain [42] will be followed. The project will be carried out following the guidelines of the Declaration of Helsinki and the Standards of Good Clinical Practice. Personal data will be processed according to the LOPD. This study has been approved by the Ethics Research Committee Provincial de Malaga (Approval number: 1216/PIA14), Spain (December 20, 2016) and has been registered in ClinicalTrials.gov with ID NCT03473704 (March 21, 2018).

All the data collected in this study will be recorded anonymously, strictly following the laws and data protection regulations in force (Law 41/2002 of November 14; Law 15/1999 of December 15).

In order to protect the confidentiality of the personal information of the participants, the following measures have been taken: all the data that can identify the participant will be kept separate from the rest of the information collected in the different questionnaires of the study; each case of the study will have an identification number that will be the one that appears in the databases; the information analysis will always be done in an aggregate way and never individually; all researchers involved in the project undertake to comply with the necessary standards to preserve the confidentiality of the information provided by the participants; personal data will be permanently unlinked from study data in order to protect the identity of the participants (data anonymization method); all study databases will be electronically protected with codes that limit access only to project researchers.

#### Procedure

The participants will be individuals with type 1 diabetes who regularly attend consultations in the Clinical Management Unit of Endocrinology and Nutrition of 2 hospitals (tertiary care) and a specialty center (secondary care) of the Andalusian Health Service of the province of Malaga (Spain) who voluntarily choose to participate in the study. The participants will not receive any type of economic or material remuneration.

Patients will be recruited through information posters placed in the visible areas of the Units, inviting them to participate in the study, either directly by sending an e-mail or through their physician. Those interested in participating will be evaluated by a psychologist from the research group who will assess the inclusion and exclusion criteria. Depressive symptoms will be evaluated with the Beck Depression Inventory-FastScreen (BDI-FS) [43] Patients with a BDI-FS score greater than or equal to 4 and less than 13 who meet the rest of the inclusion criteria and none of the exclusion criteria will sign the informed consent. All questions about the study will be answered, and the necessary aspects clarified, ensuring that the participant has understood everything he or she has read. The participants will then be prepared to begin the study and the initial assessment will be conducted.

**Fig 1:**
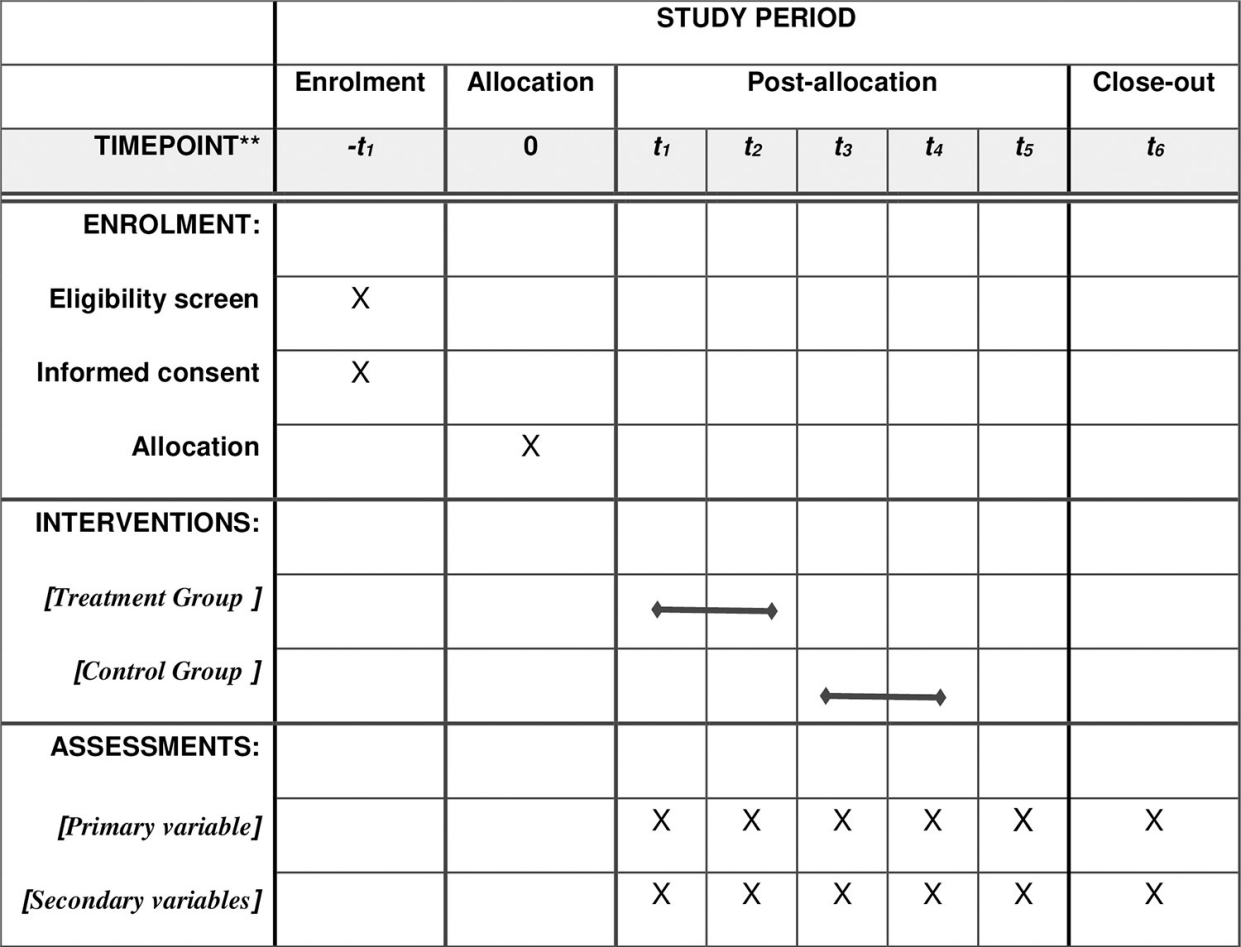
SPIRIT schedule of enrolment, intervention and assessments. Note: -t1, Eligibility screen, Informed consent; t1, baseline assessment; t2, post 8-week intervention; t3, 3-month follow-up; t4, 6-month follow-up; t5, 12-month follow-up; t6, participants complete treatment and follow-up in both the TG and the CG.

Once the sample has been randomized, the participants will be notified of the group to which they have been assigned by e-mail. Those assigned to the CG will be given encouragement while they wait to begin treatment after the TG has completed the treatment (Fig 1).

#### Intervention

The web page will include the 9 sessions that comprise the CBT-based treatment (Table 1). The sessions will be weekly. Each week, the participant will find a brief summary of the previous session and an introduction to the topic being covered, with an explanation of the new session. The participant will then be provided with the new information (the subject matter) and various examples will be included to facilitate understanding. Once the information is introduced, a summary of the key ideas of the session will be presented and a short evaluation will assess the subject’s understanding. At the end of the session, the person will have the key ideas and a self-assessment of the session. Finally, the participant will receive an explanation of the task to be completed and forwarded to the therapist with an example of how the task should be carried out. The therapist will respond and provide feedback on the completed assignment, but no personal advice will be given. Each session will need to be completed in order to have the following session available. In addition, the participant will have a bibliography of each of the topics covered. Each session will last 20–30 minutes.

**Table 1:** Treatment sessions.

Session	Topic
1	What is depression? Relationship between cognitions, behavior and emotions. Relationship between diabetes and depression.
2	Relationship between stress and diabetes. Use of relaxation and physical exercise for the management of stress and diabetes.
3	Coping in diabetes. How to face the fears associated with diabetes (short-/medium- and long-term complications).
4	Identification, definition and resolution of problems. The demands of daily self-care.
5	Pleasurable activities. Incorporation of a physically and mentally healthy lifestyle plan.
6	Cognitive restructuring. Modification of beliefs and cognitive errors in diabetes.
7	Social skills: communication style. Working towards an assertive communication style. Communication with family, friends and medical professionals.
8	Importance of support. Searching for sources of support regarding diabetes.
9	Relapse prevention.

Therapists will send e-mails to the TG participants to encourage them to continue their efforts and they will be called if there is no response to these e-mails. Halfway through the program, their opinions about it will be sought (doubts, suggestions for improvement). This is intended to motivate the participants in order to improve adherence and minimize the risk of drop out.

The CG (waiting list) participants will receive a telephone call or e-mail informing them of the next program start date. In the event that during the study an individual (CG or TG) is found to have a worsening depressive mood (severe depression or suicidal ideation), the participant would discontinue the study and his or her physician would be notified in order to refer the individual to the Clinical Management Unit of Mental Health of the Andalusian Health Service.

Protocol amendments: Important protocol modifications will be reported to the researchers, the University of Malaga and the protocol registry.

## Results assessed

The variables set out below will be collected at each assessment point, with the exception of personality, which will only be collected at the beginning of the study (Table 2).

**Table 2:** Variables evaluated in the study.

Variable	Measurement instrument	Measurement time
Sociodemographic data, habits, diabetes support and previous Mental Health treatment.	Structured interview	Baseline, post-treatment, 3 months, 6 months, 1 year
Clinical data and glycemic control	Record sheet	Baseline, post-treatment, 3 months, 6 months, 1 year
Depressive symptoms	Beck Depression Inventory FastScreen (BDI-FS, Sanz, Izquierdo & García-Vera, 2011)	Baseline, post-treatment, 3 months, 6 months, 1 year
Depression	Structured Clinical Interview for DSM-V Major Depression Episode (SCID-5-CV) (First, Williams, Karg & Spitzer, 2016).	Baseline, post-treatment, 3 months, 6 months, 1 year
Fear of hypoglycemia	Fear of Hypoglycemia questionnaire (FH-15) (Anarte et al., 2011)	Baseline, post-treatment, 3 months, 6 months, 1 year
Diabetes-related distress	Diabetes-related distress. The Spanish version (Fisher, Glasgow, Mullan, Skaff & Polonsky, 2008) of the Diabetes Distress Scale (DDS) by Polonsky et al. (2005) will be used.	Baseline, post-treatment, 3 months, 6 months, 1 year
Quality of life	Diabetes Quality of Life Questionnaire (DQOL) (The DCCT Research Group, 1988).	Baseline, post-treatment, 3 months, 6 months, 1 year
Anxiety	Anxiety through the Spanish version (Seisdedos, 1988) of the State-Trait Anxiety Inventory (Spielberger, Gorsuch & Lushene, 1982).	Baseline, post-treatment, 3 months, 6 months, 1 year
Coping	Coping with the Spanish version (Crespo & Cruzado, 1997) of the COPE Inventory (Carver, Scheier & Weintraub, 1989).	Baseline, post-treatment, 3 months, 6 months, 1 year
Treatment adherence	Diabetes Self-Care Inventory-Revised questionnaire (SCI-R). The Spanish version will be used to evaluate treatment adherence (Jansà, Vidal, Giménez, Conget, Galindo et al, 2013).	Baseline, post-treatment, 3 months, 6 months, 1 year
Personality	Personality through the Millon Clinical Multiaxial Inventory-III (MCMI-III) (Millon, Davis and Millon, 1997), Spanish version by Cardenal and Sánchez (2007)	Baseline

Through a structured interview, sociodemographic data, medication taken and healthy and harmful habits will be recorded, as well as previous Mental Health treatment and diabetes-related support.

Clinical data and glycemic control will be collected through the medical record form of the visit to the physician and the data provided by the participant.

The primary variable is depression. Depressive symptoms during the previous 2 weeks will be assessed through a self-administered 7-item questionnaire (0–3), the BDI-FS [43]. For the diagnosis of depression, the Structured Clinical Interview for Major Depression Episode of the DSM-V will be used [44].

Among the secondary variables, fear of hypoglycemia will be evaluated with the Fear of Hypoglycemia questionnaire (FH-15) [45], which consists of 15 negative items on a Likert-type scale (1–5), and diabetes-related distress using the Spanish version [46] of the Diabetes Distress Questionnaire (DDS) by Polonsky et al., [47] which has 16 Likert-type items (1–5). The DDS provides a total distress score and 4 specific scores: affective distress; distress associated with the doctor-patient relationship; regimen-related distress and interpersonal distress. Quality of life will be assessed with the Diabetes Quality of Life Questionnaire [48], which evaluates quality of life specifically in individuals with diabetes mellitus. The Spanish version [49] has 43 questions (1–5) and 4 subscales: Dissatisfaction, Impact, Social/vocational concern and Concern for future aspects. Anxiety will be addressed using the Spanish version [50] of the State-Trait Anxiety Inventory (STAI) [51]. The STAI consists of 2 scales: State Anxiety (STAI-S) and Trait Anxiety (STAI-T). Each scale includes 20 self-administered items (0–3). Coping will be evaluated with the Spanish version [52] of the COPE inventory [53], composed of 5 scales assessing problem-centered coping, another 5 scales measuring emotion-centered coping, and 3 scales measuring less useful coping responses. Adherence to treatment will be evaluated with the 15-item (1–5) Spanish version of the Diabetes Self-Care Inventory-Revised questionnaire [54]. Personality will be evaluated using the Millon Clinical Multiaxial Inventory-III Spanish version [55]. This questionnaire contains 175 items with true or false answers. It consists of 11 basic personality scales, 3 pathological characteristics scales, 7 moderate syndrome scales and 3 severe syndrome scales.

The results of the trial will be published in peer-reviewed journals and presented in conferences.

### Statistical analysis

First, a descriptive analysis will be conducted of the variables studied. For quantitative variables, measures of centralization and dispersion will be collected. For qualitative variables, a frequency analysis will be carried out.

To analyze cross-sectional and longitudinal differences, hypotheses will be contrasted at a 95% confidence level. To contrast differences between both groups (TG and CG) at baseline and post-treatment at a given time, Student’s *t*-test will be used (for strong violations of the normality assumption, the Mann-Whitney U-test) or analysis of variance (ANOVA). The effect size will be calculated using Cohen’s d.

To test whether the treatment produces differences over time (baseline, post-treatment, 3 months, 6 months and 12 months in the TG and baseline and post-treatment in the CG) in each of the groups, the *t*-test for related samples will be used (Wilcoxon signed-rank test in the event of strong violations of the normality assumption) or with an ANOVA for repeated variables and multiple regression model to control possible confounding variables (such as: age, gender, HbA1c, time with diabetes, etc.). For qualitative variables, McNemar’s test will be used.

To study the relationships between the different variables, Pearson’s correlation coefficient and linear regression models will be used for interval variables. The relationship between qualitative variables will be evaluated with the χ2 test.

Possible risk factors will be studied through logistic regression analysis (analysis of adherence to the treatment program).

The analyses will be carried out considering the subjects who complete the program. Analyses will also be carried out according to the protocol.

Analysis of the results will begin when the TG subjects have completed all of the phases of the treatment program. An improvement in the different variables will be taken into account in the following cases: significant differences in p≤0.05 and in the case of the BDI-FS when there is a decrease in the level of depressive symptomatology (moderate-mild, mild-minimum).

### Sample size calculation

We calculated the effect size using Cohen’s [56] estimates for the calculation of sample size a priori. For this, the significance level was considered to be 0.05 and the power 0.80. Concerning the effect size, according to a recent review by Uchendu and Blake [14] on CBT in adults with diabetes, there is a moderate effect size (-0.43) in depression. Therefore, in our study, a moderate effect size (0.50) will be used. With these data, a sample size of 28 participants is needed. Since the study has 2 groups, 28 participants will be included in each of the groups (56 participants in total).

## Discussion

In this article, we present the design and methodology of the WEB_TDDI1 study, developed by our research group for the treatment of mild-moderate depressive symptoms in individuals with type 1 diabetes, with the aim of providing assistance to people experiencing this problem. An easily accessible platform has been created that includes 9 treatment sessions based on CBT, the experience of previous research in this population and the previous experience of pioneering authors of this type of Web therapy for people with diabetes [29–31].

Depression in individuals with diabetes is one of the issues associated with adherence, health outcomes and costs. Although screening of these patients is recommended to identify the presence of depression and prevent affected patients from going undetected so they may be correctly treated, screening does not always ensure that the patient wants to be treated [57]. In addition, the same study reported that once the screening period had ended the number of patients referred to mental health care decreased significantly [57] Burton et al. [58] found an increase in subjects treated after screening, but this increase was small [58] Therefore, treatment options that are familiar to the patient with diabetes and are flexible in their use could help increase the percentage of subjects who are treated once detected.

We start from a treatment that is recognized as effective in the population in which it is to be applied [29–34] in depressive symptoms or depression and distress related to diabetes. However, no significant results were found regarding glycemic control, understood as the glycosylated hemoglobin (HbA1c) value. A possible explanation could be that, despite working with aspects related to the impact on the patient’s mood, changes in the treatment regimens for diabetes care are not directly addressed. This would make sense, as it is not the aim of this type of intervention. The study of other outcome variables of the web treatment on glycemic control, such as number of self-tests, episodes of hypoglycemia and hyperglycemia, and treatment adherence could add information along these lines. Moreover, other outcome variables with implications for treatment adherence, such as specific diabetes-related distress or fear of hypoglycemia, may also indicate structural changes that could influence long-term glycemic control. In addition, the number of sessions involved in treatment in previous studies has varied between 6 [32–36], 7 [37, 38] and 8 [29–31] treatment sessions. Based on previous studies and the existing relationships between depression and other variables in people with type 1 diabetes, in this study we have created 9 sessions.

Although it is known that adherence to Internet-based CBT treatment is lower than adherence when delivering the same treatment face to face [59], in the population with diabetes, there appears to be no difference between the two types of delivery [28]. Determining the variables involved in adherence to treatment will be among the data to be obtained in the present study. Accordingly, a comprehensive analysis of the medical and psychological variables involved in depression in people with diabetes, together with the variables that may affect adherence to this type of treatment in our study population, may help to provide greater insight into the effects of this treatment and the characteristics of the population in which it would be most beneficial (adherence), which are the main strengths of this study.

However, this study is not without limitations. The sample sizes of the previous studies are larger than the figure we aim to achieve in this study. Nevertheless, taking into account the rigor of the exclusion criteria and the sample size calculations described previously will enable reliable and valid conclusions to be drawn.

## Conclusions

In this article, we present the design and methodology of the WEB_TDDI1 study, which includes a specific proposal for the treatment of mild-moderate depressive symptoms in adults with type 1 diabetes delivered through a Web-based CBT program. Based on previous studies, we anticipate an improvement in depressive symptomatology with respect to the control group and to baseline status, as well as an improvement in the secondary variables studied related to depression (anxiety, diabetes-related distress, fear of hypoglycemia, coping strategies, quality of life and adherence to diabetes treatment). Thus, this program is expected to provide professionals with a tool to treat mild to moderate depressive symptomatology in people with type 1 diabetes. Additionally, an analysis of the characteristics related to adherence to this program will be carried out to help establish profiles of people who will benefit from this type of treatment.

## Supporting information

S1 Table World Health Organization trial registration data set. (DOCX)

S2 Table SPIRIT 2013 checklist: Recommended items to address in a clinical trial protocol and related documents*. (DOC)

S1 Appendix (DOCX)

S2 Appendix (DOCX)
