# Supplementary material for: Internet-based Cognitive-behavioral therapy (CBT) for depressive symptomatology in individuals with type 1 diabetes (WEB_TDDI1 study): A randomized controlled trial protocol
Source: PLoS One. 2022 Sep 20;17(9):e0274551. doi: 10.1371/journal.pone.0274551 (PMC9488778; doi:10.1371/journal.pone.0274551)
Supplement: S1 Table — (DOCX) [file pone.0274551.s001.docx]

**Supplementary Table 1.World Health Organization Trial Registration Data Set.**

| **Data category** | **Information** |
| --- | --- |
| Primary registry and trial identifying number | ClinicalTrials.gov ID: NCT03473704 |
| Date of registration in primary registry | March 21, 2018 |
| Secondary identifying numbers |  |
| Source(s) of monetary or material support | The University of Málaga has contributed to the financing of this study through its Own Plan with two projects, providing material means for the design (Proyecto Puente of the University of Málaga: Development of a telematic application for the treatment of depression in type 1 diabetes.) and development of the same (Proyecto Puente B.5 of the University of Málaga: Evaluation of the effectiveness of a new telematic program for the treatment of depression in patients with type 1 diabetes.) as well as personnel (postdoctoral contract). |
| Primary sponsor | University of Málaga |
| Secondary sponsor(s) | Biomedical Research Institute of Malaga (IBIMA) |
| Contact for public queries | Maria Teresa Anarte, PhD +34 952132994 anarte@uma.es; MónicaCarreira, PhD +34 952136697 mcarreira@uma.es. Department of Personality, Assessment and Psychological Treatment. University of Malaga, Málaga, Spain.  ] |
| Contact for scientific queries | Maria Teresa Anarte, PhD +34 952132994 [anarte@uma.es](mailto:anarte@uma.es);MónicaCarreira, PhD +34 952136697 [mcarreira@uma.es](mailto:mcarreira@uma.es)Department of Personality, Assessment and Psychological Treatment. University of Malaga, Málaga, Spain. |
| Public title | Telematic Program for the Treatment of Depression in Type 1 Diabetes. WEB_TDDI1 STUDY |
| Scientific title | Assessment of the Effectiveness of a New Telematic Program for the Treatment of Depression in Patients With Type 1 Diabetes |
| Countries of recruitment | Spain |
| Health condition(s) or problem(s) studied | Type 1 diabetes; Depression |
| Intervention(s) | The treatment group (TG) will receive the 9-week Web-based treatment, while the control group (CG) will comprise patients on the waiting list. Evaluations in the TG will be conducted at the beginning of the program (baseline), at completion and during follow-up (3, 6, and 12 months) after treatment. For ethical reasons, the CG will receive the Web-based treatment once the TG intervention has been completed. The CG will thus be assessed at the beginning and at the end of the TG treatment. At this point, the CG will begin treatment, provided the participants still meet the eligibility criteria, and will have their corresponding evaluations |
| Key inclusion and exclusion criteria | o Inclusion criteria: medical diagnosis of type 1 diabetes ≥ 1 year; over 18 years of age; psychological diagnosis of mild/moderate major depressive disorder, dysthymia or depressive symptomatology; no concomitant pharmacological treatment that could modify blood glucose values or depressive symptomatology; no previous psychological treatment; absence of the following: chronic renal failure, impaired liver function tests, active thyroid disease (except correctly substituted hypothyroidism), current pregnancy or acute ketosis decompensation at the beginning of the study; Internet access.  o Exclusion criteria: type 2 diabetes; women who are pregnant or planning to become pregnant; severe macrovascular or microvascular complications; diagnosis of major depressive disorder with risk of suicide; non-collaboration (no signed informed consent); presenting a disabling psychiatric disorder, psychosis, diagnosis of major depressive disorder or suicidal ideation; no Internet access. |
| Study type | Study Type: Interventional  Primary Purpose: Treatment  Study Phase: N/A  Interventional Study Model: Parallel Assignment  The treatment group (TG) will receive the web treatment, which consists of 9 weekly sessions, while the control group (CG) will be evaluated in the same phases as the TG. For ethical reasons, the CG will receive the web treatment once the TG intervention has been completed. There will be a follow-up at 3, 6 and 12 months.  Number of Arms: 2  Masking: Single (Care Provider)  Allocation: Randomized |
| Date of first enrolment | October 1, 2020 [Anticipated, problems with COVID-19] |
| Target sample size | 40 [Anticipated] |
| Recruitment status | Recruiting |
| Primary outcome(s) | 1. Depression  Structured Clinical Interview for Diagnostic and Statistical Manual of Mental Disorders-5 (SCID-5)  [Time Frame: Change between Pre treatment, post treatment (12 weeks), 3, 6 and 12 months.]  2. Depression symptoms  Beck Depression Inventory–Fast Screen (BDI-FS)  [Time Frame: Change between Pre treatment, post treatment (12 weeks), 3, 6 and 12 months.] |
| Key secondary outcomes | 3. Fear of Hypoglycemia  Fear of Hypoglycemia Scale (FH-15): 15 summable items assessed on a 5-point Likert scale with a range of 1–5. The  cutoff score was set at 28 points. Individuals with scores equal to or greater than 28 points will be classified as having  fear of hypoglycemia.  [Time Frame: Pre treatment, post treatment (12 weeks), 3, 6 and 12 months.]  4. Distress  Diabetes Distress Scale (DDS): 17 items measure that uses a Likert scale to score each item from 1 (no problem)  to 6 (a serious problem) during the last month. Based on four distress-related domains: emotional burden subscale,  physician-related distress subscale, regimen-related distress subscale, and diabetes-related interpersonal distress.  [Time Frame: Pre treatment, post treatment (12 weeks), 3, 6 and 12 months.]  5. Quality of Life  Diabetes Quality of Life Questionnaire (DQOL)  [Time Frame: Pre treatment, post treatment (12 weeks), 3, 6 and 12 months.]  6. Anxiety  The State-Trait Anxiety Inventory (STAI)  [Time Frame: Pre treatment, post treatment (12 weeks), 3, 6 and 12 months.]  7. Coping  Coping Inventory (COPE)  - Page 4 of 6 -  [Time Frame: Pre treatment, post treatment (12 weeks), 3, 6 and 12 months.]  8. Personality  Millon Clinical Multiaxial Inventory (MCMI-III)  [Time Frame: Pre treatment.]  9. Adherence  Self-Care Inventory-revised (SCI-R)  [Time Frame: Pre treatment, post treatment (12 weeks), 3, 6 and 12 months.]  10. Glycemic control  Glycosylated hemoglobin test (HbA1c)  [Time Frame: Pre treatment, post treatment (12 weeks), 3, 6 and 12 months.]  11. Hypoglycemia  Number of mild hypoglycemia weekly and severe hypoglycaemia in the last year.  [Time Frame: Pre treatment, post treatment (12 weeks), 3, 6 and 12 months.] |
| Key secondary outcomes | 12. Hyperglycemia  Number of hyperglycemia weekly.  [Time Frame: Pre treatment, post treatment (12 weeks), 3, 6 and 12 months.]  13. Ketone  Number of ketone test positive in the last year.  [Time Frame: Pre treatment, post treatment (12 weeks), 3, 6 and 12 months.]  14. Blood glucose self-tests  Number of blood glucose self-tests daily.  [Time Frame: Pre treatment, post treatment (12 weeks), 3, 6 and 12 months.]  15. Complications of diabetes  Collect if there is any complication of diabetes in the patient's clinical history.  [Time Frame: Pre treatment, post treatment (12 weeks), 3, 6 and 12 months.]  16. Another disease  Collect if there is any illness (other than diabetes) diagnosed by the doctor in the patient's clinical history.  [Time Frame: Pre treatment, post treatment (12 weeks), 3, 6 and 12 months.] |
