# Supplementary material for: Internet-based Cognitive-behavioral therapy (CBT) for depressive symptomatology in individuals with type 1 diabetes (WEB_TDDI1 study): A randomized controlled trial protocol
Source: PLoS One. 2022 Sep 20;17(9):e0274551. doi: 10.1371/journal.pone.0274551 (PMC9488778; doi:10.1371/journal.pone.0274551)
Supplement: S1 Appendix — (DOCX) [file pone.0274551.s003.docx]

**ESTUDIO:** Evaluación de la efectividad de un nuevo programa telemático para el tratamiento de la depresión en pacientes con diabetes 1.

**Datos del solicitante**

**Apellidos:** CARREIRA SOLER

**Nombre:** MÓNICA

**Área de conocimiento:** PERSONALIDAD, EVALUACIÓN Y TRATAMIENTO PSICOLÓGICO

**Grupo de investigación:** INVESTIGACIÓN SOBRE EL ABORDAJE INTEGRAL DE LA DIABETES

**Centro:** FACULTAD DE PSICOLOGÍA. UNIVERSIDAD DE MÁLAGA

Trabajo de investigación del plan propio de la UMA para su realización mediante contrato postdoctoral de la Universidad de Málaga concedido a Mónica Carreira Soler, cuyo grupo de investigación de la UMA de referencia es INVESTIGACIÓN SOBRE EL ABORDAJE INTEGRAL DE LA DIABETES, del que es responsable  María Teresa Anarte Ortiz

**Antecedentes**

La depresión esel trastorno psicológico con mayor morbilidad en pacientes con diabetes. La presencia de ambas enfermedades aumenta en el paciente el número de complicaciones derivadas de la diabetes, disminuye su adherencia al tratamiento de la diabetes e incrementa los costes sanitarios con respecto a las personas con diabetes que no presentan depresión. Por ello, es especialmente relevante realizar una detección precoz y un correcto tratamiento. Sin embargo, solamente un 25% de los pacientes con diabetes y depresión son identificados en su visita al médico y, por lo tanto, un 75% no tendrían acceso a un correcto tratamiento. En una reciente revisión sobre la efectividad de los tratamientos de depresión en pacientes con diabetes (van der Feltz-Cornelis et al., 2010) se confirma que el tratamiento de depresión en personas con diabetes es efectivo. Al comparar distintos tipos de tratamientos (psicoterapia, farmacoterapia y cuidados colaborativos), se encuentra que la psicoterapia combinada con educación en autocuidados para los pacientes con diabetes tipo 1 (DM1) y tipo 2 (DM2) es la que ofrece mejores resultados, tanto en variables psicológicas como en el control glucémico (Markowitz, Carper, González, Delahanty&Safren, 2012). Por otro lado, gracias al auge de las nuevas tecnologías entre la población, se plantean nuevas vías de tratamiento a través de internet, con grandes ventajas con respecto a los tratamientos tradicionales (Spek et al., 2007).

En diabetes, varios autores han diseñado programas web que han integrado terapia cognitiva-conductual (TCC) con educación diabetológica, obteniéndose resultados positivos en el estado de ánimo del paciente (Bond et al., 2010; van Bastelaar, Pouwer, Cuijpers, Twisk&Snoek, 2008; van Bastelaar, Pouwer, Cuijpers, Riper&Snoek, 2011). Sin embargo, queda pendiente el conocer qué implicaciones tiene este tipo de tratamiento a largo plazo (van Bastelaar et al, 2008).

Aunque en España se han realizado algunos estudios sobre tratamientos basados en internet, como los de Cristina Botella y Rosa Baños en pacientes con fobia y más recientemente en depresión (programa “sonreír es divertido”), o el estudio multicéntrico de Fermín Mayoral Cleries, no existen antecedentes en el ámbito de la diabetes al respecto, sólo cabe mencionar una iniciativa para informar a profesores y alumnos que conviven con niños con DM1 a través de internet, denominado “el rincón de Carol” (Salaverria y Beléndez, 2011). No obstante, no se trata de una iniciativa terapéutica, sino informativa. Además, los tratamientos web para la depresión citados aún no han demostrado su eficacia.

**Referencias:**

Bond, G.E., Burr,R.L., Wolf, F.M. &Feldt, K. (2010). The Effects of a Web-Based. Intervention on Psychosocial Well-Being Among Adults Aged 60 and Older With Diabetes. A Randomized Trial.The Diabetes Educator Online, 36, 446-445.

Salaverría, V. &Beléndez, M. (2011).Aceptación del “rincón de Carol”, un sitio web sobre la diabetes en la escuela. Avances en Diabetología, 27, 86.

vanBastelaar, K.,M.,P., Pouwer, F, Cuijpers, P., Twisk, J.W.R. &Snoek, F.J. (2008). Web-based cognitive behavioural therapy (W-CBT) for diabetes patients with co-morbid depression: Design of a randomized controlled trial. BMC Psychiatry, 8, 9.

vanBastelaar, K.M.P., Pouwer, F., Cuijpers, P., Riper, H. &Snoek, F.J. (2011). Web-based depression treatment for type 1 and type 2 diabetic patients. Diabetes Care, 34, 320-325.

van der Feltz-Cornelis, C.M., Nuyen, J., Stoop, C., Chan, J., Jacobson, A.M., Katon, W., Snoek, F., Sartorius, N. (2010). Effect of interventions for major depressive disorder and significant depressive symptoms in patients with diabetes mellitus: a systematic review and meta-analysis. General Hospital Psychiatry, 32 (4), 380-395.

**Hipótesis de partida y objetivos**

**Hipótesis:**

Se espera que el grupo tratamiento (GT) presente mejor control glucémico, sintomatología depresiva y otras variables psicológicas (menor distrés relacionado a la diabetes, desaparición/disminución del miedo a las hipoglucemias, menor ansiedad, mayor calidad de vida, mayor adherencia la tratamiento y estrategias de afrontamiento más adecuadas) que el grupo control (GC).

Objetivo 1) Aplicar un programa telemático para el tratamiento de la depresión específico para DM1 en una muestra de pacientes con DM1 y sintomatología depresiva leve-moderada de la provincia de Málaga.

- Subobjetivo 1: Determinar el nivel de sintomatología depresiva en la muestra de estudio (pacientes con DM1).

Objetivo 2) Evaluación integrada del impacto del tratamiento en las variables de estudio.

- Subobjetivo 1: Evaluar el impacto del nuevo tratamiento en la sintomatología depresiva de los pacientes.

- Subobjetivo 2: Evaluar el impacto del tratamiento en variables biomédicas (control glucémico, complicaciones de la diabetes, etc).

- Subobjetivo 3: Evaluar el impacto del tratamiento en miedo a las hipoglucemias.

- Subobjetivo 4: Evaluar el impacto del tratamiento en distrés relacionado con la diabetes.

- Subobjetivo 5: Evaluar el impacto del tratamiento en ansiedad.

- Subobjetivo 6: Analizar el impacto del tratamiento en las estrategias de afrontamiento.

- Subobjetivo 7: Analizar el impacto del tratamiento en función de la personalidad.

- Subobjetivo 8: Analizar el impacto del tratamiento en calidad de vida.

- Subobjetivo 9: Analizar el impacto del tratamiento en la adherencia al tratamiento diabetológico.

Objetivo 3) Estudiar la asociación entre sintomatología depresiva y las variables de estudio.

- Subobjetivo 1: Estudiar la asociación entre sintomatología depresiva y variables biomédicas (control glucémico, complicaciones de la diabetes, etc).

- Subobjetivo 2: Estudiar la asociación entre sintomatología depresiva y miedo a las hipoglucemias.

- Subobjetivo 3: Estudiar la asociación entre sintomatología depresiva y distrés relacionado con la diabetes.

- Subobjetivo 4: Estudiar la asociación entre sintomatología depresiva y ansiedad.

- Subobjetivo 5: Estudiar la asociación entre sintomatología depresiva y afrontamiento.

- Subobjetivo 6: Estudiar la asociación entre sintomatología depresiva y personalidad.

- Subobjetivo 7: Estudiar la asociación entre sintomatología depresiva y calidad de vida.

- Subobjetivo 8: Estudiar la asociación entre sintomatología depresiva y adherencia al tratamiento diabetológico.

Objetivo 4) Analizar los resultados de este tratamiento para cada una de las variables estudiadas durante el período de seguimiento (3, 6 y 12 meses).

**Metodología**

*Participantes*

Los participantes serán pacientes con diabetes tipo 1 (DM1) de la provincia de Málaga que cumplan los siguientes criterios:

- Criterios de inclusión: Tener un diagnóstico médico de diabetes tipo 1; ser mayor de 18 años de edad; tener diagnóstico psicológico de trastorno depresivo mayor leve/moderado, distimia o sintomatología depresiva; no tener un tratamiento farmacológico concomitante que pudiera modificar los valores de glucemia o la sintomatología depresiva; no estar en tratamiento psicológico previo; ausencia de: insuficiencia renal crónica, alteración de las pruebas de función hepática, enfermedad tiroidea activa (excepto hipotiroidismo correctamente sustituido), gestación en curso; ausencia de descompensación aguda cetósica al inicio del estudio; tener acceso a internet.
- Criterios de exclusión: diabetes tipo 2; mujeres embarazadas o planificando embarazo; complicaciones macro o microvasculares severas; diagnóstico de trastorno depresivo mayor grave con riesgo de suicidio; no colaboración (no firma de consentimiento informado); no disponer de acceso a internet; presentar algún trastorno psiquiátrico incapacitante, psicosis, diagnóstico de trastorno depresivo grave, ideas de suicidio; no disponer de acceso a internet.

*Variables*

1) Variables sociodemográficas: edad, sexo, nivel educativo, etc.

2) Variable Independiente (VI): Niveles de la VI

V.1.- Tratamiento telemático para la sintomatología depresiva en pacientes con DM1: “Plataforma web” (grupo tratamiento)

V.2.- Lista de espera (grupo control)

3) Variables dependientes (VD):

3.1.- Variables clínicas (biomédicas):

Variable principal:

- - HbA1c (hemoglobina glicosilada plasmática y capilar).

Variables secundarias:

- - Hipoglucemias leves percibidas y desapercibidas semanales (autoregistros).
  - Hiperglucemias semanales.
  - Hipoglucemias graves (episodios/ 6 meses).
  - Cetosis (episodios/mes), Cetoacidosis (episodios /6meses).
  - Número de autocontroles.
  - Complicaciones actuales de la diabetes.
  - Otro tipo de enfermedad.

3.2.-Variables psicosociales (psicométricas):

Variable principal: Depresión

Variables secundarias:

- Miedo a las hipoglucemias

- - Distrés
  - Ansiedad
  - Afrontamiento
  - Calidad de vida
  - Adherencia
  - Personalidad

La variable *Personalidad*será medida sólo al inicio del estudio en ambos grupos.El resto de variables descritas se medirán en las fases indicadas en la Tabla 1.

*Instrumentos*

1) Datos generales: Datos sociodemográficos (edad, sexo, nivel socioeducativo, ambiente familiar, hábitos saludables: tabaco, café, sueño, ejercicio, peso, otras drogas, medicamentos, etc.), medicación consumida y hábitos tóxicos. Estos datos se recogerán de las historias clínicas de los pacientes a través de una hoja de registro estructurada.

2) Datos clínicos y control glucémico:

- - HbA1c (plasmática y capilar).
  - Hipoglucemias leves percibidas (< 70 mg/dl) y desapercibidas semanales (autoregistros).
  - Hiperglucemias semanales (>250 mg/dl).
  - Hipoglucemias graves (episodios/ 6 meses).
  - Cetosis (episodios/mes), Cetoacidosis (episodios /6meses).
  - Número de autocontroles.
  - Complicaciones actuales de la diabetes.
  - Otro tipo de enfermedad.

3) Datos psicométricos:

3.1.- Depresión

a) Entrevista Clínica Estructurada para los Trastornos del Eje I del DSM-IV (SCID-1)(First et al., 1999).En este estudio, cuando se habla de depresión se hace referencia al trastorno depresivo mayor. Para realizar el diagnóstico de este trastorno se utilizó la *Entrevista Clínica Estructurada para los Trastornos del Eje I del DSM-IV* (SCID-1, First, Spitzer, Gibbon y Williams, 1999).Ésta es una entrevista destinada a establecer los diagnósticos más importantes del Eje I del DSM-IV (American PsychiatricAssociation, 1994). Está dividida en seis módulos relativamente independientes y se realiza en una única sesión. Mediante esta entrevista estructurada, se clasificaron los pacientes de este estudio según la presencia del trastorno o la ausencia del mismo, en función de los criterios DSM.

b) Inventario de Depresión de Beck (BDI-II). Se utilizará la version española del Beck DepressionInventory-II (Sanz, Perdigón & Vázquez, 2003). El BDI-II evalúa la intensidad de la sintomatología depresiva en las dos últimas semanas. Cuestionario autoadministrado con 21 items (0-3).

c) Escala de Depresión en Diabetes Tipo 1 (DDI) elaborada y validada por nuestro grupo de trabajo. Esta escala consta de 45 ítems negativos medidos en una escala Likert (1. Totalmente en desacuerdo, 2. Bastante en desacuerdo, 3. Ligeramente en desacuerdo, 4. Ni de acuerdo ni en desacuerdo, 5. Ligeramente de acuerdo, 6. Bastante de acuerdo y 7. Totalmente de acuerdo), agrupadosen siete factores (Factor 1: Síntomas; Factor 2: Abandono del cuidado de la diabetes; Factor 3: Desesperanza e Insatisfacción; Factor 4: Culpa; Factor 5: Diabetes; Factor 6: Indefensión; Factor 7: Interferencia en la vida diaria). El punto de corte está en 155 puntos.

3.2.- Miedo a las hipoglucemias

El miedo a las hipoglucemias se valoró con el cuestionario de Miedo a las Hipoglucemias “FH-15” (Anarte et al., 2011). Este instrumento consta de 15 ítems negativos, evaluados mediante una escala tipo Likert cuyos valores oscilan entre 1 y 5 (1= Nunca a 5 =Todos los días).

3.3.-Distrés

Para evaluar el nivel de estrés relacionado con la diabetes, se utilizará el Cuestionario de Distrés para Diabéticos (DDS) de Polonsky et al. (2005), el cual cuenta con 16 ítems tipo Likert, con cinco opciones de respuestas desde muy en desacuerdo (1), hasta muy de acuerdo (5), por lo que una mayor puntuación indica mayor nivel de estrés. Esta escala arroja una puntuación total de distrés y cuatro dimensiones: distrés afectivo; distrés asociado a la relación médico-paciente; distrés relacionado al régimen y distrés interpersonal.

3.4. - Calidadde vida

“Diabetes Quality of Life Questionnaire” (DQOL) (The DCCT Research Group, 1988).Este cuestionario valora calidad de vida, específicamente en personas con diabetes mellitus. La versión española (Millán, Reviego& del Campo, 2002) tiene un total de 43 preguntas. Tiene cuatro subescalas: Insatisfacción, Impacto, Preocupación social/vocacional y Preocupación por aspectos futuros. Las respuestas se cuantifican utilizando una escala Likert de 5 respuestas ordinales.

3.5.- Ansiedad

“Cuestionario de ansiedad estado-rasgo” (STAI) Para evaluar ansiedad se utilizó la versión española (Seisdedos, 1988) del StateTraitAnxietyInventory adaptada por TEA (Spielberger, Gorsuch&Lushene, 1982).El Cuestionario de Ansiedad Estado-Rasgo (STAI) consta de dos escalas de autoevaluación que miden dos conceptos independientes de la ansiedad: Estado (E) y Rasgo (R). La escala de Ansiedad-Estado (STAI-E) evalúa la situación del sujeto ante situaciones amenazantes en un momento dado, mientras que la escala de Ansiedad-Rasgo (STAI-R) supone una permanencia de ansiedad en el sujeto, evaluando una disposición a responder con elevadas tasas de ansiedad a situaciones estresantes. Cada escala consta de 20 ítems autoaplicables que se valoran en una escala de Likert (0-3).

3.6.- Afrontamiento

Escala de Afrontamiento COPE (Carver et al., 1989). Se valorará el afrontamiento realizado por los participantes a través de la versión española de la Escala de Afrontamiento COPE (Crespo & Cruzado, 1997). El cuestionario COPE (Carver, Scheier&Weintraub, 1989), consiste en 13 escalas conceptualmente diferentes. Es un cuestionario de afrontamiento multidimensional formando por cinco escalas que miden el afrontamiento centrado en el problema (afrontamiento activo, planificación, eliminación de actividades interferentes, afrontamiento restringido y búsqueda de apoyo social por razones instrumentales), cinco escalas que miden el afrontamiento centrado en la emoción (búsqueda de apoyo social por razones emocionales, desahogo de emociones, reinterpretación positiva y crecimiento, aceptación, y religión) y tres escalas que miden respuestas de afrontamiento de menor utilidad (retirada comportamental, retirada mental y negación). Esta variable será medida al comienzo del estudio, al inicio y a la finalización de los tratamientos en ambos grupos.

3.7.- Personalidad

Cuestionario Big Five (BFQ) (Caprara, Barbaranelli y Borgogni, 1995): Cuestionario con 132 elementos de respuesta múltiple (tipo Likert) para identificar las cinco dimensiones fundamentales de la personalidad humana: - Energía, Afabilidad, Tesón, Estabilidad emocional y Apertura mental. Incorpora una escala tipo 'L' para medir la deseabilidad social. Esta variable será medida sólo al comienzo del estudio.

3.8.- Adherencia

Cuestionario de Adherencia al Autocuidado de la Diabetes (SCI-R). Para evaluar adherencia al tratamiento se utilizará la versión española (Jansá, Vidal, Giménez, Conget, Galindo et al, 2013) del Diabetes Self-CareInventory- Revisedquestionnaire. El SCI-R está compuesto por 15 cuestiones que evalúan la percepción de la adherencia a las recomendaciones de autocuidado de la diabetes por parte del paciente, en el último mes. Cada cuestión se puntúa en escala Likert de 1 (nunca) a 5 (siempre).

*Diseño del estudio*

El diseño del estudio es cuasi-experimental, longitudinal aleatorizado pre-post con grupo control.

*Procedimiento*

Los pacientes con DM1 que presenten depresión (leve a moderada) serán aletorizados y asignados al azar a través de un programa informático a uno de los 2 grupos a partir del momento de inicio del estudio.Los dos grupos serán: grupo tratamiento (GT) y grupo control (GC).

El GT recibirá el tratamiento web mientras que el GC será evaluado en las mismas fases sin realizar intervención. Sin embargo, por razones éticas, el grupo control (GC) recibirá el tratamiento web una vez haya finalizado las fases de intervención y seguimiento del grupo de tratamiento (GT). Las evaluaciones serán presenciales con el objetivo de recoger los datos necesarios de una manera fiable.

De esta forma, las evaluaciones al grupo tratamiento se realizarán en los siguientes momentos: al inicio/basal, a las 9 semanas y a los 3, 6 y 12 meses después del tratamiento. El grupo control será valorado: al inicio (cuando se realice la selección aleatoria) y a la finalización del tratamiento del GT. Por consideraciones éticas, al mes de la aplicación, evaluación y seguimiento del tratamiento del GT, se llevará a cabo el tratamiento, evaluación (basal y 9 semanas) y seguimiento (3, 6 y 12 meses) del GC.

Con el objetivo de mantener la motivación del grupo control, se enviarán mensajes motivantes de continuación en el estudio vía e-mail. En el caso de detectar durante el estudio en algún paciente (GC o GT) un empeoramiento en su estado de ánimo depresivo (depresión grave o con ideas de suicidio), este paciente dejaría el estudio y sería puesto en conocimiento de su facultativo para su derivación al Servicio de Salud Mental del Servicio Andaluz de Salud (SAS).

Tabla 1. Fases de evaluación.

| Grupo Tratamiento (GT) | Grupo Control (GC) |
| --- | --- |
| Evaluación basal e inicio de tratamiento | Evaluación basal |
| Evaluación a las 9 semanas tras la realización del tratamiento | Evaluación a las 9 semanas |
| Seguimiento: evaluación a los 3 meses de terminar el tratamiento | Al mes de la finalización del GT: Evaluación basal e inicio de tratamiento |
| Seguimiento: evaluación a los 6 meses de terminar el tratamiento | Evaluación a las 9 semanas de tratamiento |
| Seguimiento: evaluación a los 12 meses de terminar el tratamiento | Seguimiento: evaluación a los 3 meses de terminar el tratamiento |
|  | Seguimiento: evaluación a los 6 meses de terminar el tratamiento |
|  | Seguimiento: evaluación a los 12 meses de terminar el tratamiento |

En la página web estarán recogidas las 9 sesiones de las que consta el tratamiento. Las sesiones serán semanales. Cada semana, el paciente se encontrará con un breve resumen de la sesión anterior y una introducción al tema tratado, donde se explicará la nueva sesión. A continuación, se le proporcionará al paciente la nueva información (el tema tratado) y se le añadirán distintos ejemplos para facilitar la comprensión. Una vez presentada la información, se presentará un resumen de las ideas claves de la sesión y una pequeña evaluación para valorar la comprensión por parte del paciente. Al finalizar la sesión, el paciente dispondrá de las ideas clave y de una autoevaluación de la sesión. Por último, se le explicará al paciente la tarea que debe realizar y enviar al terapeuta teniendo a su disposición un ejemplo de cómo realizarla. El terapeuta contestará y le dará feedback de su trabajo. Además, el paciente dispondrá de bibliografía de cada uno de los temas tratados. Cada sesión tendrá una duración de 20-30 minutos.

Basadas en la terapia cognitivo-conductual y en la educación diabetológica, además de tener en cuenta los estudios previos, las sesiones constarán de los siguientes temas:

1. ¿Qué es la depresión? Relación entre las cogniciones, el comportamiento y las emociones. Relación entre diabetes y depresión.

2. Relación entre estrés y diabetes. Utilización de la relajación y el ejercicio físico para el manejo del estrés y de la diabetes.

3. El afrontamiento en la diabetes. Cómo enfrentarse a los miedos asociados a la diabetes (complicaciones a corto/medio y largo plazo).

4. La identificación, definición y resolución de problemas. Las demandas del autocuidado diario.

5. Las actividades placenteras. Incorporación de un plan de vida saludable física y mentalmente.

6. Reestructuración cognitiva. Modificación de creencias y errores cognitivos en diabetes.

7. Habilidades sociales: el estilo de comunicación. Trabajando por un estilo de comunicación asertivo. Comunicación con la familia, amigos y profesional médico.

8. Importancia del apoyo. Búsqueda de fuentes de apoyo respecto a la diabetes.

9. Prevención de recaídas.

*Análisis de datos*

Se realizará en primer lugar un análisis descriptivo de las variables estudiadas. En el caso de variables cuantitativas, se recogerán medidas de centralización y dispersión. Para variables cualitativas, se realizará un análisis de frecuencias.

Con el objetivo de analizar diferencias transversales y longitudinales, se realizarán contrastes de hipótesis a un nivel de confianza del 95%. Para contrastar diferencias entre los dos grupos (tratamiento y control) en un momento determinado, será empleada la prueba t de Student (en caso de violaciones fuertes del supuesto de normalidad, la prueba U de Mann-Whitney). En caso de contrastes entre más de dos grupos, sería empleado el test de Kruskal-Wallis (dado el pequeño tamaño muestral de al menos uno de los grupos en ese caso).

En el caso en que las diferencias entre grupos pudieran verse afectadas por las características basales de uno de esos grupos, se realizaría un análisis de covarianza (ANCOVA) controlando los efectos de las variables que basalmente causasen diferencias.

Para contrastar si el tratamiento produce diferencias con el paso del tiempo en cada uno de los grupos, se utilizará la prueba t para muestras relacionadas (prueba de los rangos con signo de Wilcoxon en caso de violaciones fuertes del supuesto de normalidad). Si las variables objeto de análisis son cualitativas, a tal efecto se empleará el test de McNemar.

Con el fin de estudiar las relaciones entre las diferentes variables, se empleará el coeficiente de correlación de Pearson y modelos de regresión lineal, para variables de intervalo. La relación entre variables cualitativas se evaluará a partir del test de la χ2.

Con el objetivo de corroborar si existen factores de riesgo de variables dicotómicas tales como, por ejemplo, buen / mal control glucémico o presentar / no presentar síntomas depresivos, se llevarán a cabo análisis de regresión logística, para la obtención de odds ratios y riesgos relativos.

**Cronograma**

**1º año**

Fase 1: Inicio del proyecto. Planificación general. Selección de la muestra.

Fase 2: Inicio base de datos.

Fase 3: Evaluación inicial de parámetros psicológicos y clínicos (bio-médicos).

Fase 4: Inicio Tratamiento GT (evaluación GT y GC).

Fase 5: Evaluación de parámetros psicológicos y clínicos (bio-médicos) en ambos grupos tras el tratamiento aplicado en el grupo GT.

Fase 6: Inicio tratamiento en GC.

Fase 7: Evaluación de parámetros psicológicos y clínicos (bio-médicos) tras tratamiento en GC.

Fase 8: Análisis, interpretación y elaboración de resultados en ambos grupos (GT y GC).

Fase 9: Evaluación –Seguimiento (3 meses) GT.

Fase 10: Evaluación –Seguimiento (6 meses) GT.

Fase 11: Evaluación –Seguimiento (3 mes) GC.

| **Fases del estudio** | **CRONOGRAMA** | | | | | | | | | | | |
| --- | --- | --- | --- | --- | --- | --- | --- | --- | --- | --- | --- | --- |
|  | **1º TRIMESTRE** | | | **2º TRIMESTRE** | | | **3º TRIMESTRE** | | | **4º TRIMESTRE** | | |
|  | Fase 1 | Fase 1 |  |  |  |  |  |  |  |  |  |  |
|  | Fase 2 |  |  |  |  |  |  |  |  |  |  |  |
|  | Fase 3 | Fase 3 |  |  |  |  |  |  |  |  |  |  |
|  |  | Fase 4 | Fase 4 | Fase 4 |  |  |  |  |  |  |  |  |
|  |  |  |  |  | Fase 5 | Fase 5 |  |  |  |  |  |  |
|  |  |  |  |  |  | Fase 6 | Fase 6 | Fase 6 |  |  |  |  |
|  |  |  |  |  |  |  |  |  | Fase 7 | Fase 7 |  |  |
|  |  |  |  |  |  |  |  |  | Fase  8 | Fase  8 | Fase 8 |  |
|  |  |  |  |  |  |  |  | Fase 9 | Fase 9 |  |  |  |
|  |  |  |  |  |  |  |  |  |  |  | Fase 10 | Fase 10 |
|  |  |  |  |  |  |  |  |  |  |  |  | Fase 11 |

**2º año**

Fase 11: Evaluación –Seguimiento (3 mes) GC.

Fase 12: Evaluación –Seguimiento (6 meses) GC.

Fase 13: Evaluación-Seguimiento (12 meses) GT.

Fase 14: Evaluación-Seguimiento (12 meses) GC.

Fase 15: Análisis, interpretación y elaboración de resultados en ambos grupos (GT y GC): Objetivo 1.

| **Fases del estudio** | **CRONOGRAMA** | | | | | | | | | | | |
| --- | --- | --- | --- | --- | --- | --- | --- | --- | --- | --- | --- | --- |
|  | **1º TRIMESTRE** | | | **2º TRIMESTRE** | | | **3º TRIMESTRE** | | | **4º TRIMESTRE** | | |
|  | Fase 11 |  |  |  |  |  |  |  |  |  |  |  |
|  |  |  | Fase 12 | Fase 12 |  |  |  |  |  |  |  |  |
|  |  |  |  |  | Fase 13 | Fase 13 |  |  |  |  |  |  |
|  |  |  |  |  |  |  |  |  | Fase 14 | Fase 14 |  |  |
|  |  |  |  |  |  |  |  |  |  |  | Fase 15 | Fase 15 |

**3º año**

Fase 16: Análisis, interpretación y elaboración de resultados GT y GC: Objetivos 2, 3 y 4.

| **Fases del estudio** | **CRONOGRAMA** | | | | | | | | | | | |
| --- | --- | --- | --- | --- | --- | --- | --- | --- | --- | --- | --- | --- |
|  | **1º TRIMESTRE** | | | **2º TRIMESTRE** | | | **3º TRIMESTRE** | | | **4º TRIMESTRE** | | |
|  | Fase 16 | Fase 16 | Fase 16 | Fase 16 | Fase 16 | Fase 16 | Fase 16 | Fase 16 | Fase 16 | Fase 16 | Fase 16 | Fase 16 |

**4º año**

Fase 17: Redacción de resultados.

Fase 18: Presentación y difusión de resultados.*****

| **Fases del estudio** | **CRONOGRAMA** | | | | | | | | | | | |
| --- | --- | --- | --- | --- | --- | --- | --- | --- | --- | --- | --- | --- |
|  | **1º TRIMESTRE** | | | **2º TRIMESTRE** | | | **3º TRIMESTRE** | | | **4º TRIMESTRE** | | |
|  | Fase 17 | Fase 17 | Fase 17 | Fase 17 | Fase 17 | Fase 17 | Fase 17 | Fase 17 | Fase 17 | Fase 17 | Fase 17 | Fase 17 |
|  | Fase 18 | Fase 18 | Fase 18 | Fase 18 | Fase 18 | Fase 18 | Fase 18 | Fase 18 | Fase 18 | Fase 18 | Fase 18 | Fase 18 |

***Plan de difusión de resultados**

Los resultados de este proyecto serán difundidos por las siguientes vías:

1.- Publicación de al menos 4 artículos en revistas especializadas de impacto:

- - [Diabetes Technology and Therapeutics](javascript:AL_get(this,%20'jour',%20'Diabetes%20Technol%20Ther.');)
  - CurreCnt Diabetes Reports
  - Psychosomatic Medicine
  - HealthPsychology

2.-Contribución a reuniones científicas de carácter nacional e internacional (comunicaciones, pósters, symposiums, etc):

Congreso de la Sociedad Andaluza de Endocrinología y Nutrición (SAEN)

Congreso de la Asociación Europea de Evaluación Psicológica (EAPA)

Congreso de la Sociedad Española de Diabetes (SED)

Congreso de Federación Internacional de Diabetes (IDF)

Congreso de la EuropeanAssociation Diabetes (EASD)

Congreso de la ADA (ADA)

3.- Patentar el nuevo programa obtenido para el tratamiento de la sintomatología depresiva en diabetes tipo 1.

4.- Exportación del programa a nivel nacional/internacional a grupos de investigación interesados.

**Consideraciones Éticas**

El proyecto se llevará a cabo siguiendo las directrices de la Declaración de Helsinki y las Normas de Buena Práctica Clínica. Los datos de carácter personal se tratarán según la LOPD.

La documentación del estudio se presentará al Comité de Ética pertinente. El estudio comenzará en cada centro después de la aprobación/registro del Comité de Ética correspondiente.
