# Supplementary material for: Internet-based Cognitive-behavioral therapy (CBT) for depressive symptomatology in individuals with type 1 diabetes (WEB_TDDI1 study): A randomized controlled trial protocol
Source: PLoS One. 2022 Sep 20;17(9):e0274551. doi: 10.1371/journal.pone.0274551 (PMC9488778; doi:10.1371/journal.pone.0274551)
Supplement: S2 Appendix — (DOCX) [file pone.0274551.s004.docx]

**STUDY:** Effectiveness assessment of a new telematic program for the treatment of depression in patients with diabetes 1.

**Applicant details**

**Surname:** CARREIRA SOLER

**Name:** MÓNICA

**Knowledge area:** PERSONALITY, ASSESSMENT AND PSYCHOLOGICAL TREATMENT

**Research group:** RESEARCH ON THE COMPREHENSIVE APPROACH TO DIABETES

**Center:** FACULTY OF PSYCHOLOGY. MALAGA UNIVERSITY

Research work of the UMA's own plan to be carried out by means of a postdoctoral contract from the University of Malaga granted to Mónica Carreira Soler, whose reference UMA research group is INVESTIGATION ON THE INTEGRAL APPROACH TO DIABETES, for which María Teresa Anarte Ortiz is responsible.

**Background**

Depression is the psychological disorder with the highest morbidity in patients with diabetes. The presence of both diseases increases the number of complications derived from diabetes in patients, decreases their adherence to diabetes treatment and increases healthcare costs compared to people with diabetes who do not have depression. Therefore, it is especially relevant to carry out an early detection and correct treatment. However, only 25% of patients with diabetes and depression are identified during their visit to the doctor and, therefore, 75% would not have access to correct treatment. In a recent review on the effectiveness of depression treatments in patients with diabetes (van der Feltz-Cornelis et al., 2010) it is confirmed that the treatment of depression in people with diabetes is effective. When comparing different types of treatments (psychotherapy, pharmacotherapy and collaborative care), it is found that psychotherapy combined with self-care education for patients with type 1 (DM1) and type 2 (DM2) diabetes is the one that offers the best results, both in psychological variables such as glycemic control (Markowitz, Carper, González, Delahanty & Safren, 2012). On the other hand, thanks to the rise of new technologies among the population, new treatment routes are being proposed through the internet, with great advantages over traditional treatments (Spek et al., 2007).

In diabetes, several authors have designed web programs that have integrated cognitive-behavioral therapy (CBT) with diabetes education, obtaining positive results in the patient's mood (Bond et al., 2010; van Bastelaar, Pouwer, Cuijpers, Twisk & Snoek, 2008; van Bastelaar, Pouwer, Cuijpers, Riper & Snoek, 2011). However, it is still pending to know what implications this type of treatment has in the long term (van Bastelaar et al, 2008).

Although in Spain there have been some studies on internet-based treatments, such as those by Cristina Botella and Rosa Baños in patients with phobia and more recently in depression (“smiling is fun” program), or the multicenter study by Fermín Mayoral Cleries, no There are antecedents in the area of ​​diabetes in this regard, it is only worth mentioning an initiative to inform teachers and students who live with children with DM1 through the internet, called “Carol's corner” (Salaverria and Beléndez, 2011). However, it is not a therapeutic initiative, but an informative one. Also, the web-based depression treatments cited have yet to be proven effective.

**References:**

Bond, G.E., Burr,R.L., Wolf, F.M. & Feldt, K. (2010). The Effects of a Web-Based. Intervention on Psychosocial Well-Being Among Adults Aged 60 and Older With Diabetes. A Randomized Trial.The Diabetes Educator Online, 36, 446-445.

Salaverría, V. & Beléndez, M. (2011).Aceptación del “rincón de Carol”, un sitio web sobre la diabetes en la escuela. Avances en Diabetología, 27, 86.

van Bastelaar, K.,M.,P., Pouwer, F, Cuijpers, P., Twisk, J.W.R. & Snoek, F.J. (2008). Web-based cognitive behavioural therapy (W-CBT) for diabetes patients with co-morbid depression: Design of a randomized controlled trial. BMC Psychiatry, 8, 9.

van Bastelaar, K.M.P., Pouwer, F., Cuijpers, P., Riper, H. & Snoek, F.J. (2011). Web-based depression treatment for type 1 and type 2 diabetic patients. Diabetes Care, 34, 320-325.

van der Feltz-Cornelis, C.M., Nuyen, J., Stoop, C., Chan, J., Jacobson, A.M., Katon, W., Snoek, F., Sartorius, N. (2010). Effect of interventions for major depressive disorder and significant depressive symptoms in patients with diabetes mellitus: a systematic review and meta-analysis. General Hospital Psychiatry, 32 (4), 380-395.

**Starting hypothesis and objectives**

**Hypothesis:**

The treatment group (GT) is expected to present better glycemic control, depressive symptoms and other psychological variables (less distress related to diabetes, disappearance / decrease of fear of hypoglycemia, less anxiety, higher quality of life, greater adherence to treatment and more adequate coping strategies) than the control group (CG).

Objective 1) To apply a telematic program for the treatment of depression specific for DM1 in a sample of patients with DM1 and mild-moderate depressive symptoms in the province of Malaga.

- Sub-objective 1: Determine the level of depressive symptoms in the study sample (patients with DM1).

Objective 2) Integrated evaluation of the impact of the treatment on the study variables.

- Sub-objective 1: Evaluate the impact of the new treatment on the depressive symptoms of patients.

- Sub-objective 2: Evaluate the impact of the treatment on biomedical variables (glycemic control, complications of diabetes, etc.).

- Sub-objective 3: Evaluate the impact of treatment on fear of hypoglycemia.

- Sub-objective 4: Evaluate the impact of treatment in diabetes-related distress.

- Sub-objective 5: Evaluate the impact of treatment on anxiety.

- Sub-objective 6: Analyze the impact of treatment on coping strategies.

- Sub-objective 7: Analyze the impact of treatment based on personality.

- Sub-objective 8: Analyze the impact of treatment on quality of life.

- Sub-objective 9: Analyze the impact of treatment on adherence to diabetes treatment.

Objective 3) To study the association between depressive symptoms and the study variables.

- Sub-objective 1: Study the association between depressive symptoms and biomedical variables (glycemic control, complications of diabetes, etc.).

- Sub-objective 2: Study the association between depressive symptoms and fear of hypoglycemia.

- Sub-objective 3: Study the association between depressive symptoms and diabetes-related distress.

- Sub-objective 4: Study the association between depressive symptoms and anxiety.

- Sub-objective 5: Study the association between depressive symptoms and coping.

- Sub-objective 6: Study the association between depressive symptoms and personality.

- Sub-objective 7: Study the association between depressive symptoms and quality of life.

- Sub-objective 8: Study the association between depressive symptoms and adherence to diabetes treatment.

Objective 4) To analyze the results of this treatment for each of the variables studied during the follow-up period (3, 6 and 12 months).

**Methodology**

Participants

The participants will be patients with type 1 diabetes (DM1) from the province of Malaga who meet the following criteria:

o Inclusion criteria: Have a medical diagnosis of type 1 diabetes; Be over 18 years old; have a psychological diagnosis of mild / moderate major depressive disorder, dysthymia or depressive symptoms; not having a concomitant pharmacological treatment that could modify blood glucose values ​​or depressive symptoms; not be in previous psychological treatment; absence of: chronic renal failure, impaired liver function tests, active thyroid disease (except correctly substituted hypothyroidism), ongoing pregnancy; absence of acute ketosis decompensation at baseline; have internet access.

o Exclusion criteria: type 2 diabetes; pregnant women or planning pregnancy; severe macro or microvascular complications; diagnosis of serious major depressive disorder with risk of suicide; non-collaboration (no signing of informed consent); not have internet access; present any disabling psychiatric disorder, psychosis, diagnosis of severe depressive disorder, suicidal ideation; not having internet access.

Variables

1) Sociodemographic variables: age, sex, educational level, etc.

2) Independent Variable (VI): VI levels

V.1.- Telematic treatment for depressive symptoms in patients with DM1: “Web platform” (treatment group)

V.2.- Waiting list (control group)

3) Dependent variables (DV):

3.1.- Clinical variables (biomedical):

Main variable:

- HbA1c (plasma and capillary glycosylated hemoglobin).

Secondary variables:

o Mild hypoglycemia perceived and unnoticed weekly (self-registration).

o Weekly hyperglycemia.

o Severe hypoglycemia (episodes / 6 months).

o Ketosis (episodes / month), Ketoacidosis (episodes / 6 months).

o Number of self-checks.

o Current complications of diabetes.

o Another type of illness.

3.2.-Psychosocial variables (psychometric):

Primary variable: Depression

Secondary variables:

- Fear of hypoglycemia

- Distress

- Anxiety

- Coping

- Quality of life

- Adhesion

- Personality

Personality variable will be measured only at the beginning of the study in both groups. The rest of the variables described will be measured in the phases indicated in Table 1.

Instruments

1) General data: Sociodemographic data (age, sex, socio-educational level, family environment, healthy habits: tobacco, coffee, sleep, exercise, weight, other drugs, medications, etc.), medication consumed and toxic habits. These data will be collected from the patients' medical records through a structured record sheet.

2) Clinical data and glycemic control:

o HbA1c (plasma and capillary).

o Mild hypoglycemia perceived (<70 mg / dl) and unnoticed weekly (self-registration).

o Weekly hyperglycemia (> 250 mg / dl).

o Severe hypoglycemia (episodes / 6 months).

o Ketosis (episodes / month), Ketoacidosis (episodes / 6 months).

o Number of self-checks.

o Current complications of diabetes.

o Another type of illness.

3) Psychometric data:

3.1.- Depression

a) Structured Clinical Interview for DSM-IV Axis I Disorders (SCID-1) (First et al., 1999). In this study, when talking about depression, it refers to major depressive disorder. The DSM-IV Structured Clinical Interview for Axis I Disorders (SCID-1, First, Spitzer, Gibbon & Williams, 1999) was used to diagnose this disorder. This is an interview designed to establish the most important DSM-IV Axis I diagnoses (American Psychiatric Association, 1994). It is divided into six relatively independent modules and takes place in a single session. Using this structured interview, the patients in this study were classified according to the presence or absence of the disorder, based on the DSM criteria.

b) Beck Depression Inventory (BDI-II). The Spanish version of the Beck Depression Inventory-II (Sanz, Perdigón & Vázquez, 2003) will be used. The BDI-II assesses the intensity of depressive symptoms in the last two weeks. Self-administered questionnaire with 21 items (0-3).

c) Type 1 Diabetes Depression Scale (DDI) developed and validated by our working group. This scale consists of 45 negative items measured on a Likert scale (1. Strongly disagree, 2. Strongly disagree, 3. Slightly disagree, 4. Neither agree or disagree, 5. Slightly agree, 6. Quite a bit of agree and 7. Totally agree), grouped into seven factors (Factor 1: Symptoms; Factor 2: Abandonment of diabetes care; Factor 3: Hopelessness and dissatisfaction; Factor 4: Guilt; Factor 5: Diabetes; Factor 6: Helplessness ; Factor 7: Interference in daily life). The cutoff point is at 155 points.

3.2.- Fear of hypoglycemia

The fear of hypoglycemia was assessed with the FH-15 Fear of Hypoglycemia questionnaire (Anarte et al., 2011). This instrument consists of 15 negative items, evaluated using a Likert-type scale whose values ​​range between 1 and 5 (1 = Never to 5 = Every day).

3.3.-Distress

To assess the level of stress related to diabetes, the Diabetic Distress Questionnaire (DDS) by Polonsky et al. (2005), which has 16 Likert-type items, with five response options ranging from strongly disagree (1) to strongly agree (5), so a higher score indicates a higher level of stress. This scale gives a total distress score and four dimensions: affective distress; distress associated with the doctor-patient relationship; regime-related distress and interpersonal distress.

3.4. - Quality of life

"Diabetes Quality of Life Questionnaire" (DQOL) (The DCCT Research Group, 1988). This questionnaire assesses quality of life, specifically in people with diabetes mellitus. The Spanish version (Millán, Reviego & del Campo, 2002) has a total of 43 questions. It has four subscales: Dissatisfaction, Impact, Social / vocational concern and Concern for future aspects. The responses are quantified using a Likert scale of 5 ordinal responses.

3.5.- Anxiety

“State-trait anxiety questionnaire” (STAI) The Spanish version (Seisdedos, 1988) of the State Trait Anxiety Inventory adapted by TEA (Spielberger, Gorsuch & Lushene, 1982) was used to assess anxiety. The State-Trait Anxiety Questionnaire (STAI) consists of two self-assessment scales that measure two independent concepts of anxiety: State (E) and Trait (R). The Anxiety-State scale (STAI-E) assesses the subject's situation in threatening situations at a given moment, while the Anxiety-Trait scale (STAI-R) assumes a permanence of anxiety in the subject, evaluating a disposition to respond with high rates of anxiety to stressful situations. Each scale consists of 20 self-applied items that are assessed on a Likert scale (0-3).

3.6.- Coping

COPE Coping Scale (Carver et al., 1989). The coping carried out by the participants will be assessed through the Spanish version of the COPE Coping Scale (Crespo & Cruzado, 1997). The COPE questionnaire (Carver, Scheier & Weintraub, 1989), consists of 13 conceptually different scales. It is a multidimensional coping questionnaire consisting of five scales that measure problem-centered coping (active coping, planning, eliminating interfering activities, restricted coping, and seeking social support for instrumental reasons), five scales that measure coping centered on the problem. emotion (seeking social support for emotional reasons, release of emotions, positive reinterpretation and growth, acceptance, and religion) and three scales that measure less useful coping responses (behavioral withdrawal, mental withdrawal, and denial). This variable will be measured at the beginning of the study, at the beginning and at the end of the treatments in both groups.

3.7.- Personality

Big Five Questionnaire (BFQ) (Caprara, Barbaranelli and Borgogni, 1995): Questionnaire with 132 multiple response items (Likert type) to identify the five fundamental dimensions of human personality: - Energy, Affability, Tendency, Emotional stability and Mental openness . It incorporates an 'L' type scale to measure social desirability. This variable will be measured only at the beginning of the study.

3.8.- Adhesion

Diabetes Self-Care Adherence Questionnaire (SCI-R). To assess adherence to treatment, the Spanish version (Jansá, Vidal, Giménez, Conget, Galindo et al, 2013) of the Diabetes Self-Care Inventory-Revised questionnaire will be used. The SCI-R is made up of 15 questions that assess the patient's perception of adherence to diabetes self-care recommendations in the last month. Each question is scored on a Likert scale from 1 (never) to 5 (always).

Study design

The study design is quasi-experimental, longitudinal randomized pre-post with a control group.

Procedure

Patients withDM1 who present with depression (mild to moderate) will be randomized and randomized through a computer program to one of the 2 groups from the moment the study begins. The two groups will be: treatment group (GT) and control group (CG).

The GT will receive the web treatment while the CG will be evaluated in the same phases without performing any intervention. However, for ethical reasons, the control group (CG) will receive the web treatment once the intervention and follow-up phases of the treatment group (GT) have been completed. The evaluations will be face-to-face in order to collect the necessary data in a reliable way.

In this way, evaluations of the treatment group will be carried out at the following times: at baseline / baseline, at 9 weeks and at 3, 6 and 12 months after treatment. The control group will be evaluated: at the beginning (when the random selection is made) and at the end of the GT treatment. For ethical considerations, a month after the application, evaluation and follow-up of the GT treatment, the treatment, evaluation (baseline and 9 weeks) and follow-up (3, 6 and 12 months) of the CG will be carried out.

In order to maintain the motivation of the control group, motivating messages will be sent to continue the study via e-mail. In the case of detecting during the study in a patient (CG or GT) a worsening in their depressed mood (severe depression or suicidal thoughts), this patient would leave the study and be brought to the attention of their doctor for referral to the Mental Health Service of the Andalusian Health Service (SAS).

Table 1. Evaluation phases.

| Treatment Group (GT) | Control Group (CG) |
| --- | --- |
| Baseline evaluation and start of treatment | Baseline evaluation |
| Evaluation at 9 weeks after completion of treatment | Evaluation at 9 weeks |
| Follow-up: evaluation 3 months after finishing treatment | One month after GT completion: Baseline evaluation and start of treatment |
| Follow-up: evaluation at 6 months after finishing the treatment | Evaluation at 9 weeks of treatment |
| Follow-up: evaluation 12 months after finishing treatment | Follow-up: evaluation 3 months after finishing treatment |
|  | Follow-up: evaluation 6 months after finishing treatment |
|  | Follow-up: evaluation 12 months after finishing treatment |

The 9 sessions that comprise the treatment will be listed on the website. Sessions will be weekly. Each week, the patient will find a brief summary of the previous session and an introduction to the topic discussed, where the new session will be explained. Next, the patient will be given the new information (the topic covered) and different examples will be added to facilitate understanding. Once the information is presented, a summary of the key ideas of the session and a short evaluation will be presented to assess understanding by the patient. At the end of the session, the patient will have the key ideas and a self-evaluation of the session. Finally, the patient will be explained the task to be carried out and sent to the therapist with an example of how to do it available. The therapist will answer and give you feedback on her work. In addition, the patient will have a bibliography of each of the topics discussed. Each session will last 20-30 minutes.

Based on cognitive-behavioral therapy and diabetes education, in addition to taking into account previous studies, the sessions will consist of the following topics:

1. What is depression? Relationship between cognitions, behavior and emotions. Relationship between diabetes and depression.

2. Relationship between stress and diabetes. Use of relaxation and physical exercise for the management of stress and diabetes.

3. Coping with diabetes. How to face the fears associated with diabetes (short / medium and long term complications).

4. The identification, definition and resolution of problems. The demands of daily self-care.

5. Pleasant activities. Incorporation of a physically and mentally healthy life plan.

6. Cognitive restructuring. Modification of beliefs and cognitive errors in diabetes.

7. Social skills: communication style. Working for an assertive communication style. Communication with family, friends and medical professional.

8. Importance of support. Search for sources of support regarding diabetes.

9. Prevention of relapses.

Analysis of data

First, a descriptive analysis of the variables studied will be carried out. In the case of quantitative variables, measures of centralization and dispersion will be collected. For qualitative variables, a frequency analysis will be performed.

In order to analyze cross-sectional and longitudinal differences, hypothesis tests will be carried out at a confidence level of 95%. To contrast differences between the two groups (treatment and control) at a given time, the Student's t test will be used (in case of strong violations of the assumption of normality, the Mann-Whitney U test). In case of contrasts between more than two groups, the Kruskal-Wallis test would be used (given the small sample size of at least one of the groups in this case).

In the event that the differences between groups could be affected by the baseline characteristics of one of these groups, an analysis of covariance (ANCOVA) would be performed, controlling for the effects of the variables that caused differences at baseline.

To test whether the treatment produces differences over time in each of the groups, the t-test will be used for related samples (Wilcoxon signed rank test in case of strong violations of the assumption of normality). If the variables under analysis are qualitative, the McNemar test will be used for this purpose.

In order to study the relationships between the different variables, Pearson's correlation coefficient and linear regression models will be used for interval variables. The relationship between qualitative variables will be evaluated from the χ2 test.

In order to corroborate whether there are risk factors for dichotomous variables such as, for example, good / bad glycemic control or presenting / not presenting depressive symptoms, logistic regression analyzes will be carried out to obtain odds ratios and relative risks.

**Schedule**

**1st year**

Phase 1: Start of the project. General planning. Sample selection.

Phase 2: Database start.

Phase 3: Initial evaluation of psychological and clinical parameters (bio-medical).

Phase 4: Initiation of GT Treatment (GT and CG evaluation).

Phase 5: Evaluation of psychological and clinical parameters (bio-medical) in both groups after the treatment applied in the GT group.

Phase 6: Start treatment in CG.

Phase 7: Evaluation of psychological and clinical parameters (bio-medical) after treatment in CG.

Phase 8: Analysis, interpretation and elaboration of results in both groups (GT and CG).

Phase 9: Evaluation - Follow-up (3 months) GT.

Phase 10: Evaluation - Follow-up (6 months) GT.

Phase 11: Evaluation - Follow-up (3 months) CG.

| **Phases of the study** | **Schedule** | | | | | | | | | | | |
| --- | --- | --- | --- | --- | --- | --- | --- | --- | --- | --- | --- | --- |
|  | **1st Quarter** | | | **2ndQuarter** | | | **3rdQuarter** | | | **4thQuarter** | | |
|  | Phase 1 | Phase 1 |  |  |  |  |  |  |  |  |  |  |
|  | Phase 2 |  |  |  |  |  |  |  |  |  |  |  |
|  | Phase 3 | Phase 3 |  |  |  |  |  |  |  |  |  |  |
|  |  | Phase 4 | Phase 4 | Phase 4 |  |  |  |  |  |  |  |  |
|  |  |  |  |  | Phase 5 | Phase 5 |  |  |  |  |  |  |
|  |  |  |  |  |  | Phase 6 | Phase 6 | Phase 6 |  |  |  |  |
|  |  |  |  |  |  |  |  |  | Phase 7 | Phase 7 |  |  |
|  |  |  |  |  |  |  |  |  | Phase  8 | Phase  8 | Phase 8 |  |
|  |  |  |  |  |  |  |  | Phase 9 | Phase 9 |  |  |  |
|  |  |  |  |  |  |  |  |  |  |  | Phase 10 | Phase 10 |
|  |  |  |  |  |  |  |  |  |  |  |  | Phase 11 |

**2nd year**

Phase 11: Evaluation - Follow-up (3 months) CG.

Phase 12: Evaluation - Follow-up (6 months) CG.

Phase 13: Evaluation-Follow-up (12 months) GT.

Phase 14: Evaluation-Follow-up (12 months) CG.

Phase 15: Analysis, interpretation and elaboration of results in both groups (GT and CG): Objective 1.

| **Phases of the study** | **Schedule** | | | | | | | | | | | |
| --- | --- | --- | --- | --- | --- | --- | --- | --- | --- | --- | --- | --- |
|  | **1st Quarter** | | | **2nd Quarter** | | | **3rd Quarter** | | | **4th Quarter** | | |
|  | Phase 11 |  |  |  |  |  |  |  |  |  |  |  |
|  |  |  | Phase 12 | Phase 12 |  |  |  |  |  |  |  |  |
|  |  |  |  |  | Phase 13 | Phase 13 |  |  |  |  |  |  |
|  |  |  |  |  |  |  |  |  | Phase 14 | Phase 14 |  |  |
|  |  |  |  |  |  |  |  |  |  |  | Phase 15 | Phase 15 |

**3rd year**

Phase 16: Analysis, interpretation and elaboration of GT and CG results: Objectives 2, 3 and 4.

| **Phases of the study** | **Schedule** | | | | | | | | | | | |
| --- | --- | --- | --- | --- | --- | --- | --- | --- | --- | --- | --- | --- |
|  | **1st Quarter** | | | **2nd Quarter** | | | **3rd Quarter** | | | **4th Quarter** | | |
|  | Phase 16 | Phase 16 | Phase 16 | Phase 16 | Phase 16 | Phase 16 | Phase 16 | Phase 16 | Phase 16 | Phase 16 | Phase 16 | Phase 16 |

**4th year**

Phase 17: Drafting of results.

Phase 18: Presentation and dissemination of results. *

| **Phases of the study** | **Schedule** | | | | | | | | | | | |
| --- | --- | --- | --- | --- | --- | --- | --- | --- | --- | --- | --- | --- |
|  | **1st Quarter** | | | **2nd Quarter** | | | **3º TRIMESTRE** | | | **4th Quarter** | | |
|  | Phase 17 | Phase 17 | Phase 17 | Phase 17 | Phase 17 | Phase 17 | Phase 17 | Phase 17 | Phase 17 | Phase 17 | Phase 17 | Phase 17 |
|  | Phase 18 | Phase 18 | Phase 18 | Phase 18 | Phase 18 | Phase 18 | Phase 18 | Phase 18 | Phase 18 | Phase 18 | Phase 18 | Phase 18 |

*** Results dissemination plan**

The results of this project will be disseminated in the following ways:

1.- Publication of at least 4 articles in specialized impact magazines:

- Diabetes Technology and Therapeutics

- Current Diabetes Reports

- Psychosomatic Medicine

- Health Psychology

2.- Contribution to national and international scientific meetings (communications, posters, symposiums, etc.):

Congress of the Andalusian Society of Endocrinology and Nutrition (SAEN)

Congress of the European Association for Psychological Assessment (EAPA)

Congress of the Spanish Diabetes Society (SED)

Congress of the International Diabetes Federation (IDF)

Congress of the European Association Diabetes (EASD)

ADA Congress (ADA)

3.- Patent the new program obtained for the treatment of depressive symptoms in type 1 diabetes.

4.- Export of the program at a national / international level to interested research groups.

**Ethical Considerations**

The project will be carried out following the guidelines of the Declaration of Helsinki and the Standards of Good Clinical Practice. Personal data will be processed according to the LOPD.

The study documentation will be submitted to the relevant Ethics Committee. The Study will begin at each center after approval / registration by the Committee of corresponding Ethics.
